# Labor, with the Hymen Unbroken

**Published:** 1859-10

**Authors:** John S. Beale

**Affiliations:** Paddington-green


### *Labor, with the Hymen Unbroken.*

To the Editor of the Lancet—*Sir:* The report of your correspondent's cases of syphilis with the hymen intact, induces me to trouble you with a short record of three cases of labor in which the hymen was in the same condition. The patients were married. The first was of middling stature, robust, and eighteen years of age; the husband of average height. The second was a little woman, of slight build; the husband thin, and not more than five feet three inches high. In the third case the relative proportions of man and wife were the same as in the first. In all the cases passage of the fœtal head destroyed the membrane, without its offering any impediment to the completion of labor. In each case the hymen was situated at the orifice and not, as sometimes happens, more internally.

Dr. Wm. Hunter had the body of a young woman (brought for dissection) opened, and discovered a small fœtus, although the signs of virginity were strongly marked.—(Vide Dr. Rigby's "Midwifery," p. 52.)

The above cases bear interest in a medico-legal point of view, showing that sexual congress may be repeated, pregnancy ensue, and continue for the full period without destruction of the membrane. The three instances occurred during a period of seventeen years, and are selected from 2500 midwifery cases.

I am sir, your obedient servant,

JOHN S. BEALE, M. R. C. S.

Paddington-green, 1859. [*Lancet.*]

